# Analysis of the diagnostic and prognostic value of Peripheral blood mononuclear cell microRNA-9-5p in patients with sepsis in the intensive care unit

**DOI:** 10.3389/fcimb.2025.1509496

**Published:** 2025-04-22

**Authors:** Haoming Ye, Ruojue Wang, Qiang Ji, Qianru Li, Jinquan Liang, Miao Liu, Limian Cao, Min Shao

**Affiliations:** ^1^ Department of Critical care Medicine, the First Affiliated Hospital of Anhui Medical University, Hefei, Anhui, China; ^2^ Department of Microbiology and Parasitology, School of Basic Medical Sciences, Anhui Medical University, Hefei, China; ^3^ Center for Big Data and Population Health of Institute of Health and Medicine (IHM), Anhui Medical University, Hefei, China

**Keywords:** sepsis, peripheral blood mononuclear cell, microRNA-9-5p, diagnosis, prognosis prediction

## Abstract

**Objective:**

To investigate the diagnostic and prognostic value of miR-9-5p in peripheral blood mononuclear cells in sepsis patients.

**Methods:**

Differentially expressed miR-9-5p in sepsis were screened from a database and available literature. Subsequently, iBMDM cell validation was conducted and the expression level of miR-9-5p in peripheral blood mononuclear cells was determined using RT-qPCR in 69 sepsis patients and 30 non-sepsis patients with infections, 24 hours after ICU admission. A control group that comprised 35 healthy individuals, matched for age and sex, was set up from those who agreed to provide blood samples laboratory analysis.

**Results:**

On admission to the ICU, the levels of miR-9-5p were significantly higher in sepsis patients (10.13 [3.21, 24.94]) than in non-sepsis patients with infections (2.08 [1.68, 3.18]) and healthy controls (0.86 [0.36, 1.88]) (p < 0.001). The miR-9-5p levels were positively correlated with the severity of the disease as indicated by the SOFA score (r=0.656, P<0.001). The AUC of miR-9-5p in predicting sepsis, septic shock, and in-hospital death was 0.825, 0.821, and 0.845, respectively.

**Conclusion:**

Elevated expression levels of miR-9-5p in peripheral blood mononuclear cells are significantly associated with an increased risk of sepsis and septic shock, and also indicate a higher risk of organ dysfunction.

## 1 Introduction

Sepsis is considered a major cause of high mortality and morbidity among ICU patients (Chiu and Legrand, 2021), and according to the sepsis 3.0 definition, it is defined as life-threatening organ dysfunction caused by dysregulated host response to infection (Shankar-Hari et al., 2016). The past decade has witnessed significant progress in the development of sepsis treatments which has resulted in the improvement of medical guidelines based on new evidence and ICU monitoring and treatment technologies (Evans et al., 2021). Nevertheless, the current mortality rate associated with sepsis remains high (Schlapbach et al., 2024). This underscores the need to develop more effective strategies to improve the diagnosis of sepsis and implement timely intervention (Sinha et al., 2018).

Macrophages can regulate immune response and occurrence of inflammation in sepsis which is dependent on their polarization state (Chen et al., 2021). Further, it has been shown that macrophage polarization and apoptosis are regulated by microRNAs (Essandoh et al., 2016; Mohapatra et al., 2021). MicroRNA-9-5p is a key microRNA differentiating M1 and M2 polarized macrophages (Lu et al., 2016). MicroRNA-9-5p has been implicated in synovial tissue inflammation by regulating the AMPK signaling pathway and inhibiting SIRT1 expression (Wang et al., 2021). A study by Jie Zhen found that the Smad2/miR-9/ANO1 regulatory loop participates in LPS-induced sepsis in mice (Zhen et al., 2019).Previous studies have suggested that microRNAs are novel biomarkers in the diagnosis and treatment of sepsis (Bindayna, 2024; Xiao et al., 2024). miR-9 plays a significant role in sepsis by regulating macrophage polarization and immune response. However, there have been no reports on the clinical value of miR-9-5p expression levels in monocyte-macrophages in sepsis patients.

## 2 Methods

### 2.1 Subjects

**Figure 1:**
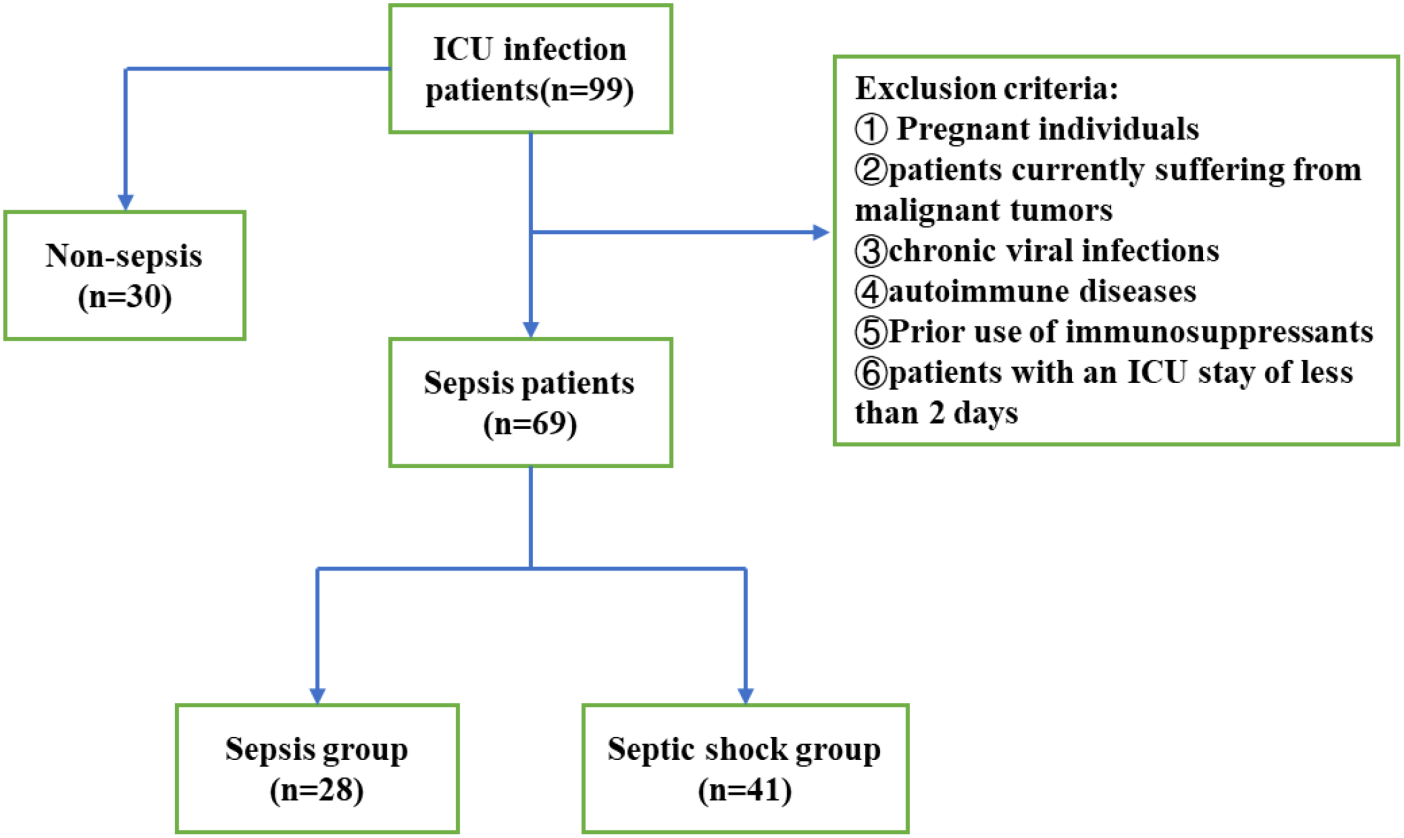
Flow chart for screening patients with sepsis.

Ninety-nine patients with sepsis who were admitted to the Department of Critical Care Medicine of the First Affiliated Hospital of Anhui Medical University from June 2022 to September 2023 were divided to two groups: sepsis group (n ¼ 51) and septic shock group (n ¼ 58). The inclusion criteria were: (1) Age 18 years old; (2) Length of stay in ICU > 2 days; (3) The diagnosis of sepsis and septic shock conforms to the international consensus on the definition of Sepsis3.0 published in April 2016, septic shock is defined as hypotension after full fluid resuscitation (mean blood pressure 65 mmHg under pressor maintenance) and serum lactic acid > 2 mmol/L (Chiu and Legrand, 2021); (4) signed the informed consent form to be enrolled in the study. In addition, the following exclusion criteria were applied: pregnant patients, patients who have recently experienced an acute heart attack (Lu et al., 2012), and those with malignant tumors; as well as patients with conditions that may affect their baseline immune status, such as autoimmune diseases, chronic viral infections, and any history of long-term immunosuppressant therapy. (**Figure 1**). The study was approved by the Ethics Committee for Clinical Research of the First Affiliated Hospital of Anhui Medical University (approval number PJ2024-02-38).

Vital signs (blood pressure, heart rate, respiratory rate, and body temperature), routine laboratory test results (creatinine, bilirubin, platelet count, C-reactive protein (CRP), Procalcitonin (PCT), hemoglobin, hematocrit, sodium, potassium, white blood cell count and blood culture), blood gas analysis (pH, lactate (Lac), PaO_2_, PaCO_2_, bicarbonate (HCO_3_), and base excess (BE)), Sequential Organ Failure Assessment (SOFA) score, Acute Physiology and Chronic Health Evaluation (APACHE) II score and personal information (age, sex) were collected. A summary of participants characteristics are show in the **Supplementary Tables 1**-**3**.

### 2.2 Sepsis model

Six- to eight-weeks old C57BL/6 male mice were purchased from Anhui Medical University (Hefei, China). They were randomly assigned to following two groups: sham surgery and sepsis. Mice in the sepsis group underwent cecal ligation and puncture (CLP) to induce sepsis, a classical mode. Mice in the sham group only underwent laparotomy. Briefly, mice were anesthetized with 2% pentobarbital and maintained at 37°C using a heating pad. A 1-cm midline abdominal incision was made following skin disinfection. To induce moderate sepsis, the cecum was ligated at its midpoint and punctured with a 21-gauge needle. A small amount of stool was squeezed out through the puncture site. The cecum was placed back into the abdomen, followed by closure of the peritoneum, fasciae and abdominal musculature with a sterile 6–0 silk suture. For mice that underwent operation, 50 ml/kg normal saline was injected subcutaneously. All mice experiments were approved by the Animal Research Ethics Society of Anhui Medical University.

### 2.3 Cell lines and cell cultures

The immortalized bone marrow-derived macrophages (iBMDM) were purchased from the Cell Bank of Chinese Academy of Sciences (Shanghai, China), and passed in DMEM (Gibco, NY, USA) enriched with 10% fetal bovine serum at 37 °C in a 5% CO_2_ humidified incubator. The cells were identified through short tandem repeats profiling and mycoplasma contamination was determined using the Cell Culture Contamination Detection Kit (Thermo Fisher). The iBMEM cells were seeded in 10-cm dishes into which 10 mL of the medium was added. Adherent cells were stimulated with 1 μg/mL LPS (Sigma-Aldrich, L6529) for 12 h following a medium change. Subsequently, five million cells per group were harvested for total RNA extraction. Each experiment was performed in triplicate. Three independent repeat experiments were conducted separately under the same experimental conditions to ensure the reproducibility and reliability of the results.

### 2.4 RNA isolation and real-time quantitative polymerase chain reaction

Peripheral blood mononuclear cells were isolated using the peripheral blood mononuclear cell kit (Solarbio, Beijing, China) following the manufacturer’s protocol. Mice liver tissues were obtained by literature format (Lu et al., 2012). Total RNA was extracted from the peripheral blood mononuclear cells and mouse liver using TRIzol reagent (Invitrogen Life Technologies, Carlsbad, CA, USA) to determine their transcript responses. Total RNA was quantified and purified using the NanoDrop ND-2000 spectrophotometer (Thermo Scientific, Waltham, MA). RT-qPCR was performed by using the 2× Universal SYBR Green Fast qPCR Mix (Nanjing Nuoweizan Biotechnology Co. Q111, LTD, China) and gene specific primers (**Supplementary Table 4**) in a CFX96 Real-time PCR detection system (Bio-Rad Laboratories GmbH, Munich, Germany).

### 2.5 Statistical analysis

Data normality was assessed using the Kolmogorov-Smirnov test. For normally distributed data, descriptive statistics (x ® ± SD) were calculated, and group differences were analyzed using the t-test. Descriptive indicators Data that did not follow normal distribution were expressed as the median and interquartile range (Q1-Q3) and the Mann-Whitney U test was used to compare groups. Qualitative data were presented as the frequency and percentage (n (%)), and chi-square test was used to analyze differences between groups. The receiver operating characteristic ROC) was developed to explore sensitivity and specificity, and Spearman correlation analysis was employed to determine correlation between expression levels of miR-9-5P and the clinical data.

For multivariable analysis, variables that showed significant associations in the univariate analysis, as well as those considered clinically important, were included to ensure the clinical plausibility of the model. We used stepwise backward regression to construct the model and employed the Hosmer-Lemeshow goodness-of-fit test to evaluate the model’s fit, with p > 0.05 considered indicative of a good fit. All data were processed and analyzed using IBM SPSS Statistics 26.0 (IBM Corporation, Armonk, NY, USA), R software (4.3.1), and GraphPad Prism 9.0. All cell-based experiments were performed in triplicate, and statistical comparisons between groups were conducted using an independent sample t-test. P<0.05 was considered statistically significant.

## 3 Results

### 3.1 Bioinformatics analysis of differential expression of miR-9-5p

**Figure 2:**
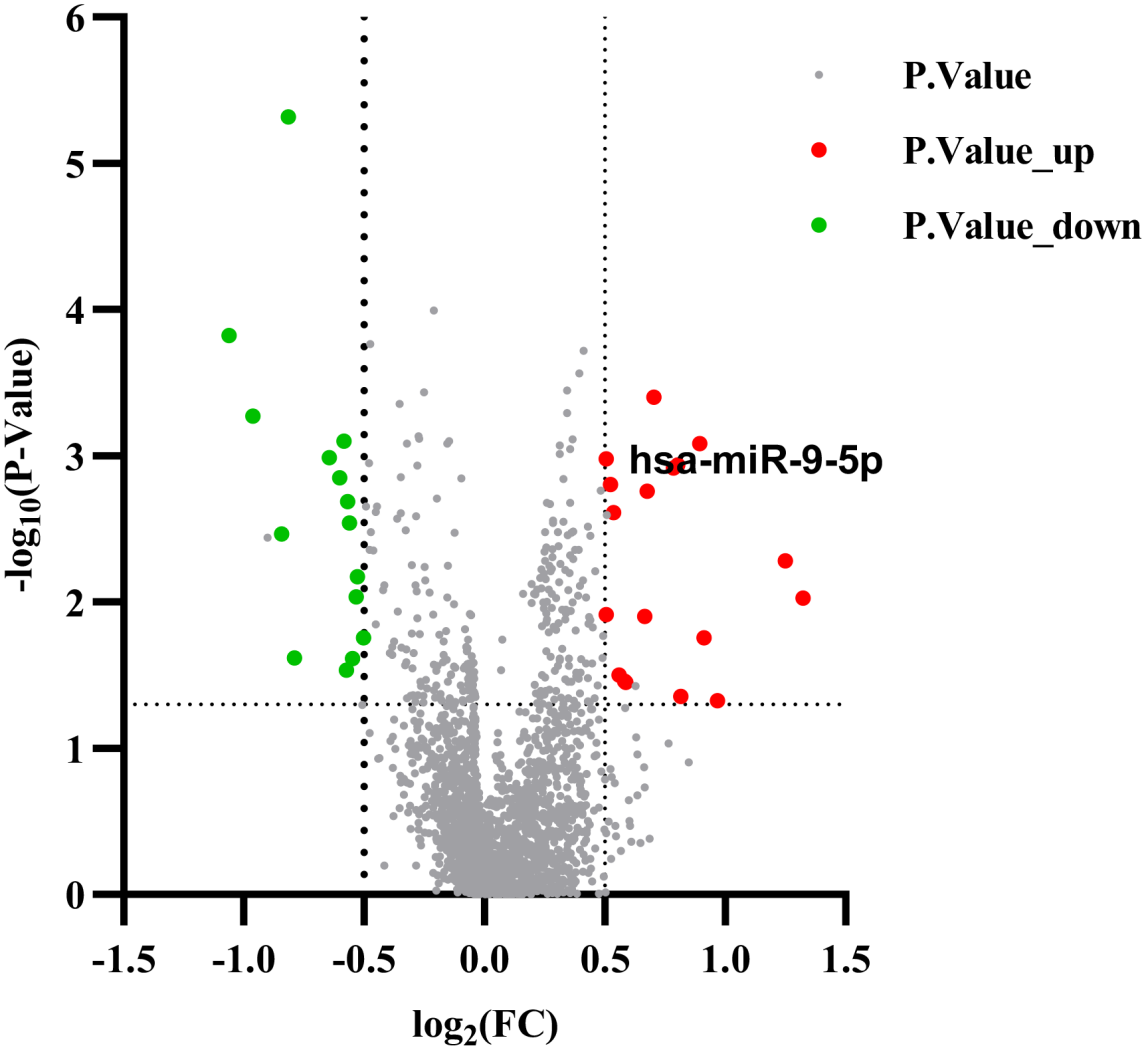
Differential microRNA Volcano Plot.

The gene expression data were analyzed on the GEO database through the NCBI portal, which collects and stores high-throughput gene expression data. The GSE152371 dataset was retrieved and processed. A volcano map was constructed using the Prism software (**Figure 2**) to display the differential microRNA, applying the selection criteria of |log₂FC| > 0.5 and FDR < 0.05). The analysis identified 18 up-regulated genes, including miR-9-5p, miR-125a-3p, miR-155-5p, and 15 down-regulated genes, such as miR-23b-3p, miR-149-5p, and miR-203a-3p. The fold change of miR-9-5p was 1.88, with p < 0.01.

**Figure 3:**
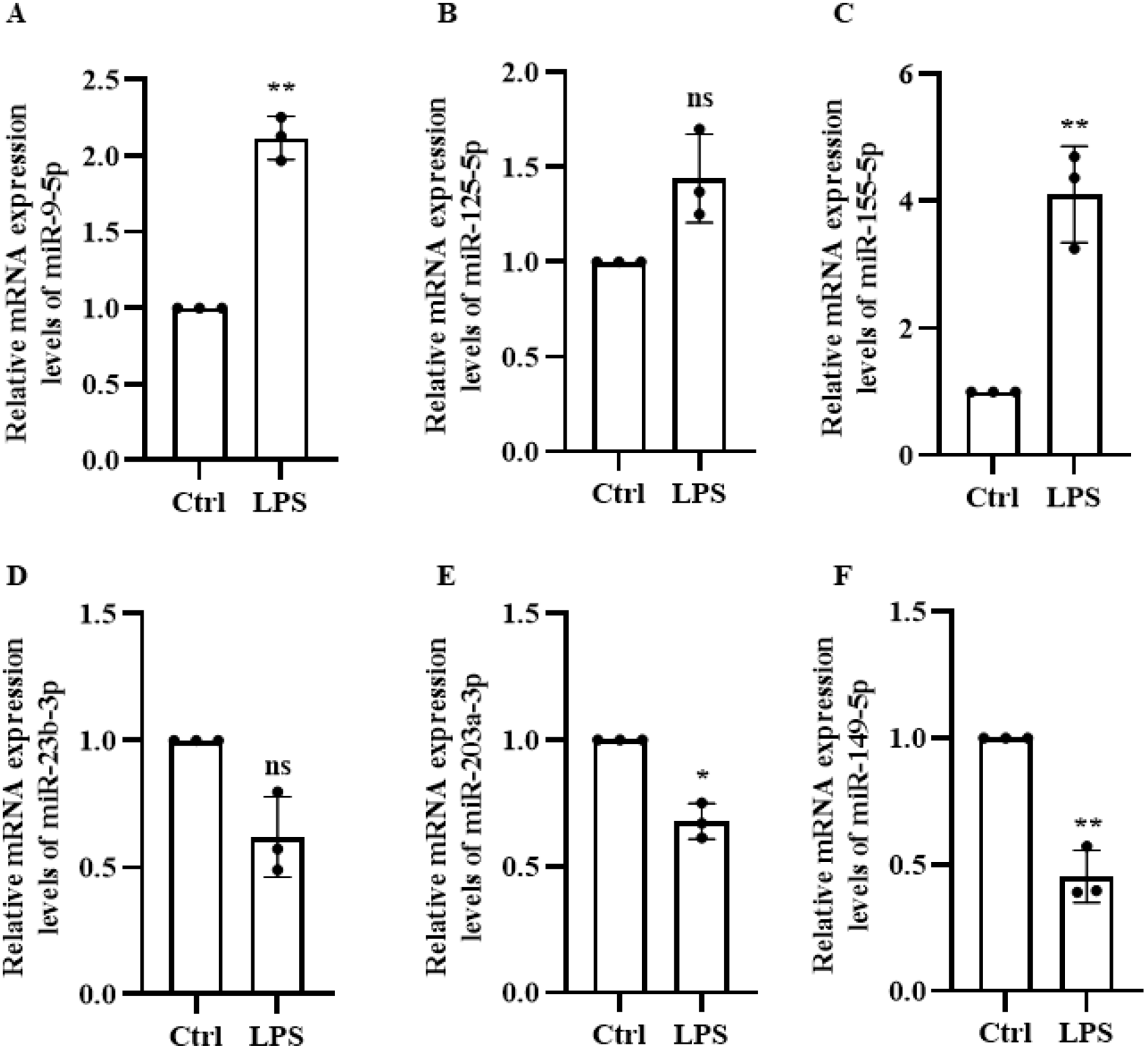
LPS-Induced Expression of six microRNAs in Macrophages. **(A)** miR-9-5p **(B)** miR-125-5p **(C)** miR-155-5p **(D)** miR-23-3p **(E)** miR-203-3p **(F)** miR-149-5p. * p < 0.05, ** p < 0.01, *** p < 0.001, ns, not significant (p ≥ 0.05).

A literature review identified six genes associated with sepsis, which were then further analyzed in a constructed lipopolysaccharide-treated mouse iBMDM macrophage inflammation model to investigate their corresponding microRNAs. **Figure 3** shows that there was no significant difference in expression level of miR-125 and miR-23b. Considering the prevailing evidence for the role of miR-155 in tumors, inflammation, and other diseases, we selected miR-9-5p which exhibited elevated differential expression in human and mouse based on homologous sequencing analysis. Its expression was highly stable during cell validation. Therefore, miR-9-5p was selected for further experiments.

### 3.2 miR-9-5p mRNA expression levels significantly upregulated in sepsis

The expression level of miR-9-5p in peripheral blood mononuclear cells from the sepsis group, non-sepsis infection group, and control group was quantified and the results are shown in **Table 1** The level of miR-9-5p in sepsis patients was significantly higher compared with that in the control group (10.13[3.21-23.94] vs. 0.86[0.36-1.88]). In addition, the expression level of miR-9-5p was significantly higher in sepsis patients relative to the non-sepsis infection group, with a median fold change of 2.08[1.68-3.18]. Moreover, the level of miR-9-5p expression showed significant differences among the three groups, with the highest expression in the sepsis group, followed by the infection without sepsis group, and the lowest in the healthy control group. This indicates the importance of infection in miR-9-5p expression.

**Table 1:** The expression of miR-9-5P in the sepsis group, the non-sepsis infection group, and the control group.

Group	Median	Interquartile Range
Control	0.86	0.36-1.88
Non-sepsis infection	2.08	1.68-3.18
Sepsis	10.13	3.21-24.94

**Figure 4:**
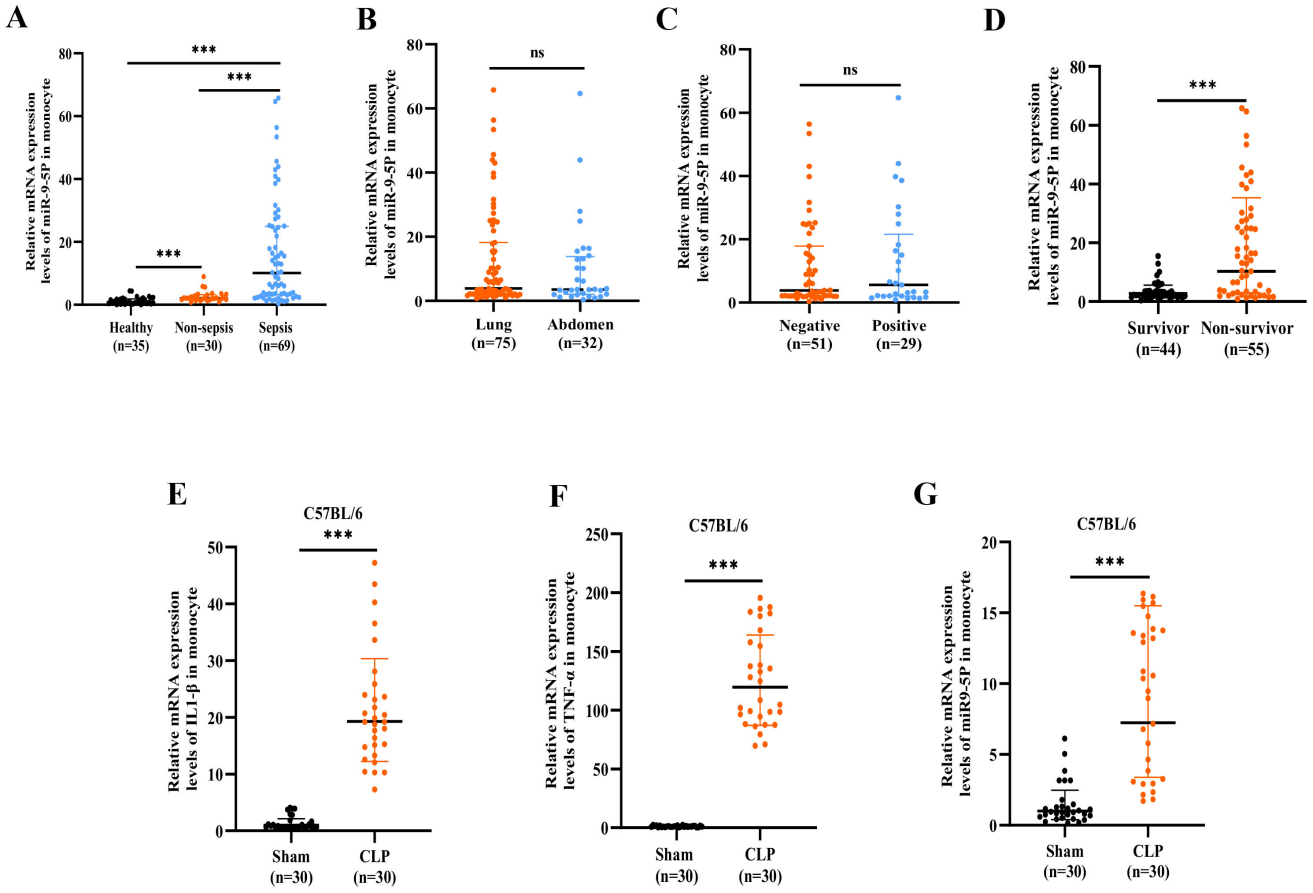
The mRNA expression level of microRNA-9-5p was significantly upregulated in sepsis. **(A)** Relative expression of microRNA-9-5p in sepsis, non-sepsis infection, and healthy controls. **(B)** The relative expression levels of microRNA-9-5p in the Gram-negative bacteria group and the Gram-positive bacteria group were cultured. **(C)** Relative expression of microRNA-9-5p in lung infection group and abdominal infection group. **(D)** Relative expression of microRNA-9-5p in survivor and non-survivor. **(E)** Relative mRNA expression levels of IL1-β in mouse monocytes(CLP, referring to the Cecal Ligation and Puncture model). **(F)** Relative mRNA expression levels of TNF-α in mouse monocytes. **(G)** Relative mRNA expression levels of microRNA-9-5p in mouse monocytes. *** p < 0.001, ns, not significant (p ≥ 0.05).

The analysis, shown in **Figures 4B, C**, compares miR-9-5p expression levels across different infection etiologies and sites of infection. The data reveal no statistically significant differences in miR-9-5p levels between positive and negative bacterial cultures or between lung and abdominal infections.

The miR-9-5p level in peripheral blood mononuclear cells was significantly higher in the non-survival group compared to the survival group (14.06 [3.35-27.93] vs. 2.32 [1.75-3.61]) (**Table 2**, **Figure 4D**). Subsequently, we developed a cecal ligation and puncture model using 6–8-week-old C57BL/6 mice, and blood samples were collected at 24 hours for q-PCR analysis. The mRNA levels of IL-1β (**Figure 4D**) and TNF-α (**Figure 4E**) in the CLP group were significantly elevated compared to the sham group, confirming the successful construction of the model. Additionally, the miR-9-5p mRNA level in the monocytes of the CLP group was significantly higher compared to levels in the sham group (**Figure 4F**), indicating that miR-9-5p mRNA levels in mouse sepsis models are upregulated compared to sham groups, which is consistent with clinical observations.

**Table 2:** The expression of miR-9-5P in the survivor and the non-survivor group.

Group	Median	Interquartile Range
Survivor	2.32	1.75-3.61
Non-survivor	14.06	3.35-27.93

### 3.3 Correlation of peripheral blood mononuclear cell miR-9-5p levels with clinical indicators

**Figure 5:**
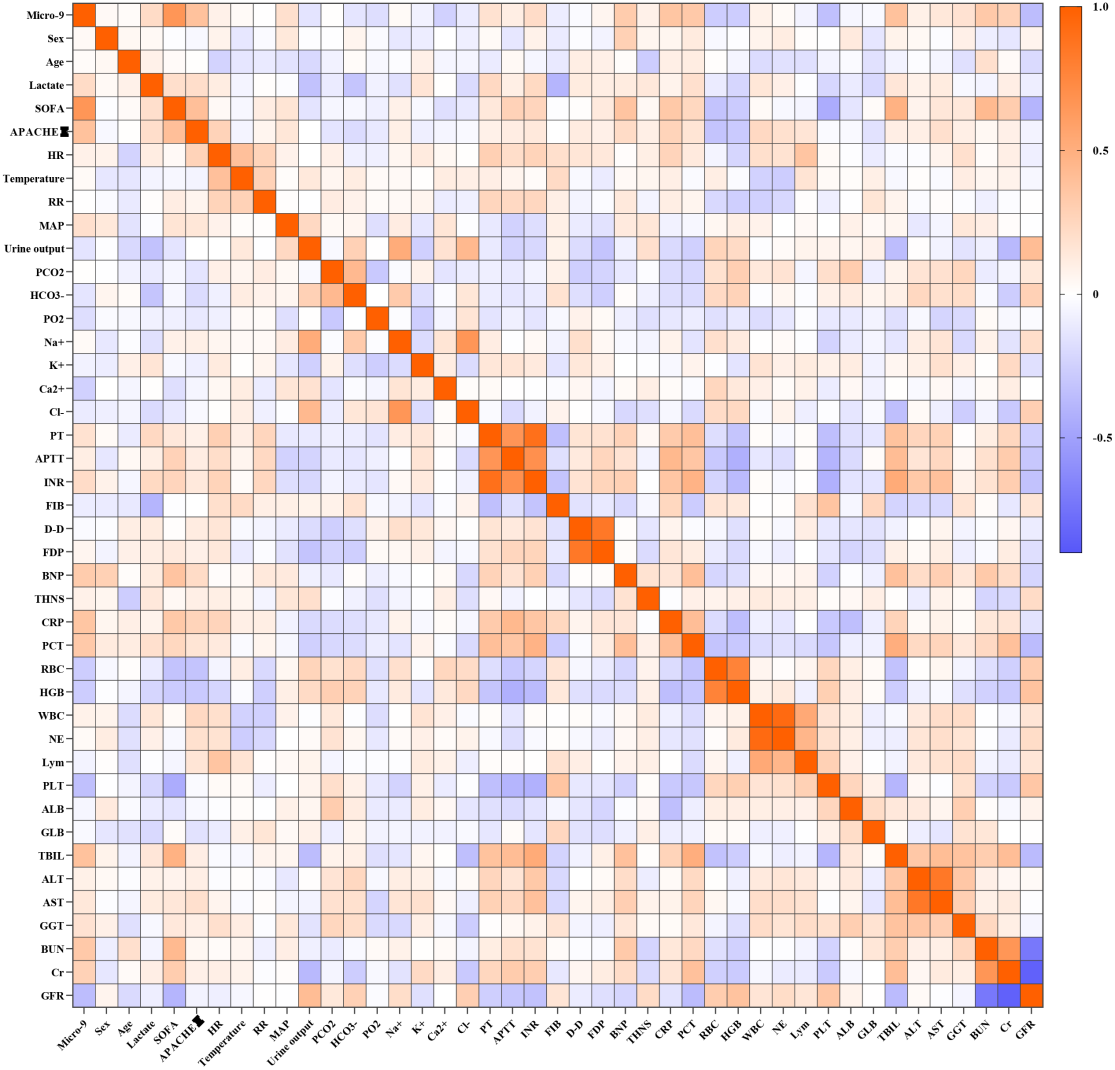
Correlation heatmap between the expression level of miR-9-5P in peripheral blood mononuclear cells and clinical indicators.

**Figure 6:**
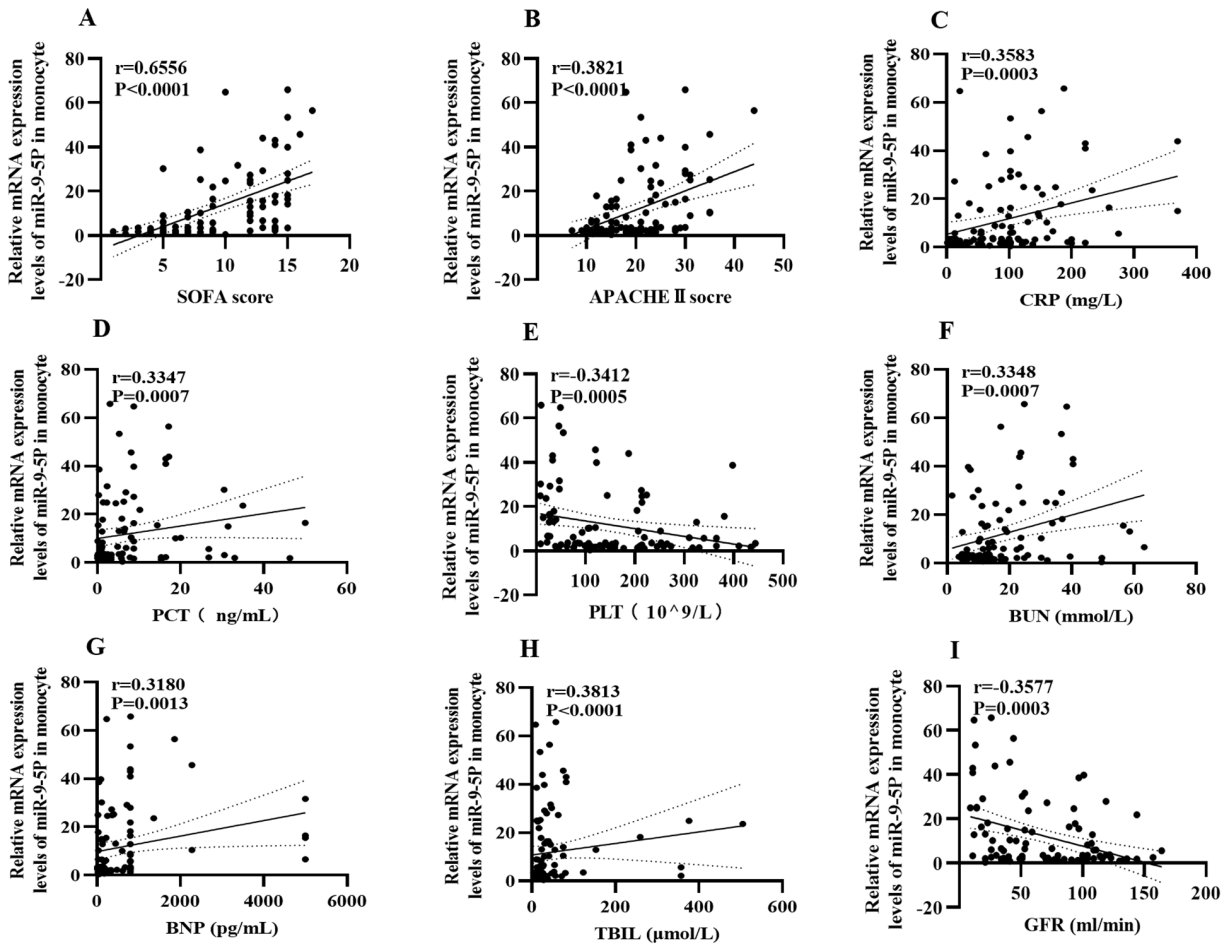
Correlation heatmap between the expression level of microRNA-9-5p in peripheral blood mononuclear cells and clinical indicators. **(A)** SOFA **(B)** APACHEII **(C)** CRP **(D)** PCT **(E)** PLT **(F)** BUN **(G)** BNP **(H)** Tbil **(I)** eGFR.

To investigate the relationship between miR-9-5p mRNA levels and clinical indicators during the sepsis progression, we analyzed the correlation between miR-9-5p levels in monocytes and various clinical indicators (**Figure 5**). It was observed that miR-9-5p levels in peripheral blood mononuclear cells were positively correlated with several clinical indicators: SOFA score (r=0.655, P<0.001; **Figure 6A**), APACHE II score (r=0.382, P<0.001; **Figure 6B**), BNP (**Figure 6G**), CRP (r=0.318, P=0.0013; **Figure 6C**), PCT (r=0.3583, P<0.001; **Figure 6D**), Tbil (r=0.328, P<0.001; **Figure 6H**), and BUN (r=0.335, P<0.001; **Figure 6F**). Conversely, miR-9-5p levels were negatively correlated with PLT (r=-0.341, P<0.001; **Figure 6E**) and eGFR (r=-0.358, P<0.001; **Figure 6I**).

### 3.4 Correlation of peripheral blood mononuclear cell miR-9-5p levels with disease severity

**Figure 7:**
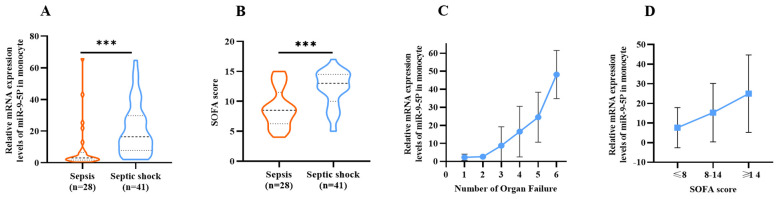
Correlation Between microRNA-9-5p Expression Level and SOFA Score Relative expression of microRNA-9-5p in peripheral blood mononuclear cells in sepsis group and septic shock group. **(B)** Comparison of SOFA score between sepsis group and septic shock group. **(C)** Relative expression of microRNA-9-5p in peripheral blood mononuclear cells increased with the increase of organ failure. **(D)** Stratification according to SOFA score. ***: p < 0.001.

The SOFA score is often utilized to evaluate the degree of organ dysfunction in sepsis and directly reflects disease severity. A high SOFA score indicates poor prognosis, and a SOFA score greater than 2 reflects organ dysfunction. A scatter plot with error bars was developed for using the number of organ failures and the relative expression level of miR-9-5p (**Figure 7C**). Analysis of the plot demonstrated a positive association between the levels of miR-9-5p and the number of failed organs. **Figure 7D** further illustrates the relative expression levels of miR-9-5p across different stratified groups based on the median SOFA score of 8 with an interquartile range from 8 to 14. Notably, there were significant differences in the miR-9-5p level among the groups.

### 3.5 Diagnostic value of miR-9-5p levels in peripheral blood mononuclear cells for sepsis

**Figure 8:**
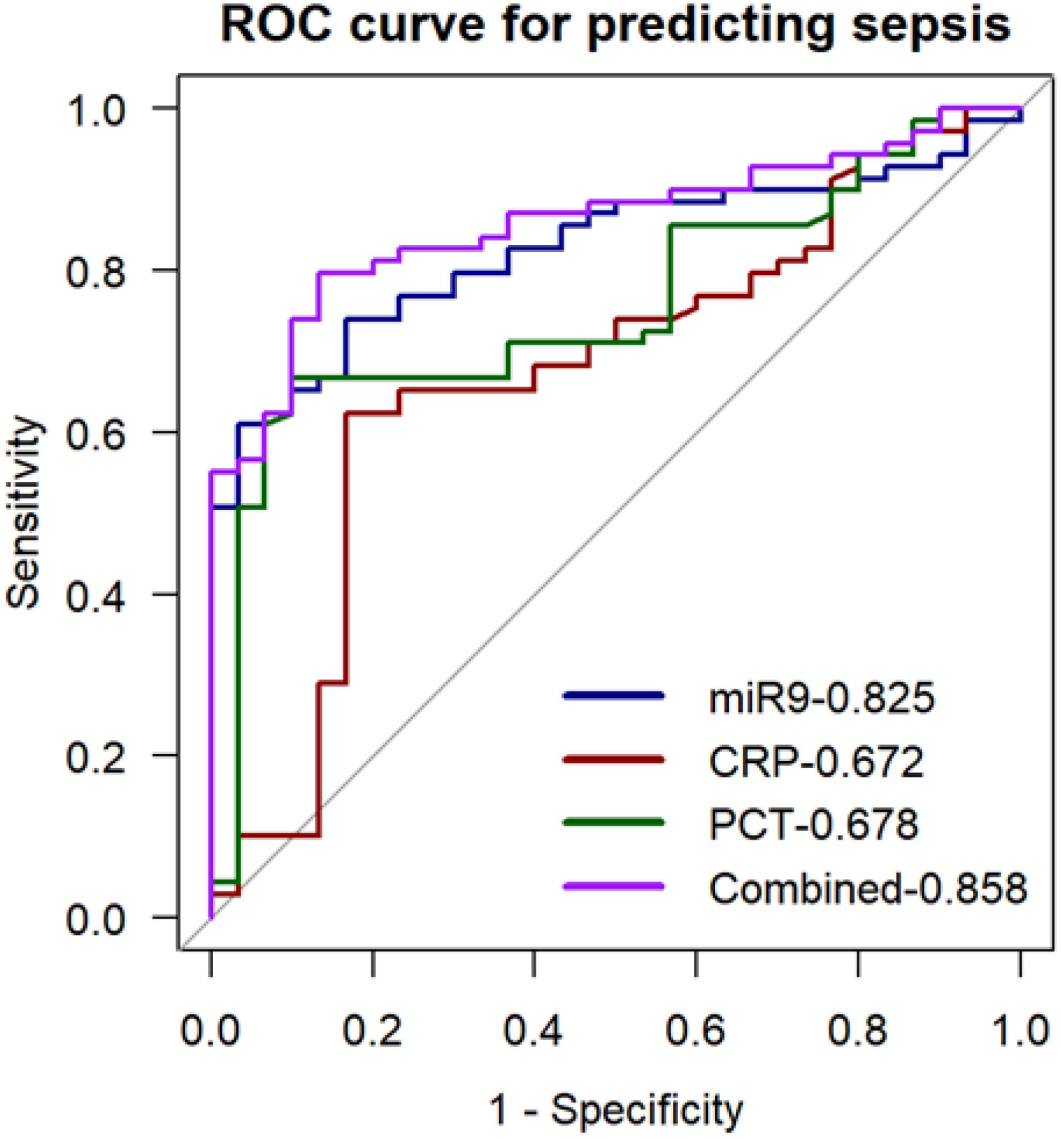
ROC Curve for predicting sepsis with the expression level of microRNA-9-5p in peripheral blood mononuclear cells.

In further experiments, we explored whether miR-9-5p levels in peripheral blood mononuclear cells may have good diagnostic value in sepsis utilizing the ROC curve and AUC based on previous results. The ROC curve analysis for miR-9-5p levels in the ICU, presented in **Table 3**, demonstrates its diagnostic value for sepsis. As shown in **Figure 8**, the diagnostic accuracy of miR-9-5p significantly surpassed that of CRP and PCT, with AUC values of 0.672 and 0.678, respectively (P < 0.001). The ROC curve derived from the combination of all three markers yielded an AUC of 0.858, which was not statistically different from the diagnostic performance of miR-9-5p alone.

**Table 3:** ROC Curve for Predicting Sepsis with the Expression Level of miR-9-5P.

Cut-off	AUC	95% CI	P	Sens	Spec	PPV	NPV
5.915	0.825	0.745-0.905	<0.001	0.609	0.967	0.977	0.518

Sens stands for sensitivity; Spec stands for specificity; PPV is the positive predictive value; NPV is the negative predictive value.

### 3.6 Diagnostic value of miR-9-5p levels in peripheral blood mononuclear cells for septic shock

**Figure 9:**
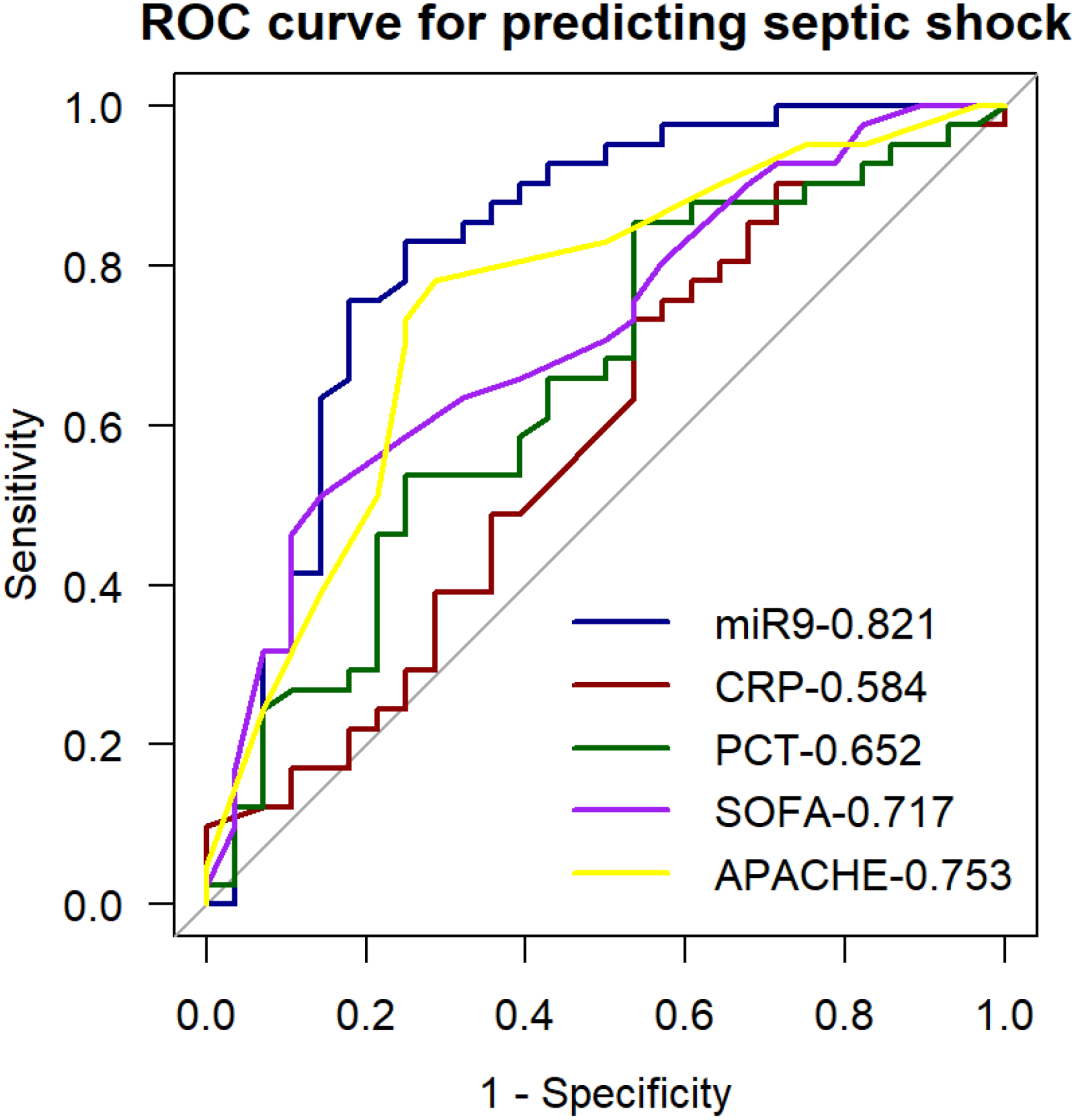
ROC curve for predicting septic shock with the expression level of microRNA-9-5p in peripheral blood mononuclear cells.

Results of the univariate ROC curve analysis demonstrated the predictive value of miR-9-5p for septic shock (**Table 4**). Multi-index ROC curves were plotted to explore the predictive performance for septic shock (**Figure 9**). The prediction accuracy of miR-9-5p in septic shock patients was statistically better than the SOFA score, CRP, PCT, and APACHE II scores(P < 0.05).

**Table 4:** ROC Curve for Predicting Septic Shock with the Expression Level of miR-9-5P.

Cut-off	AUC	95% CI	P	Sens	Spec	PPV	NPV
6.065	0.821	0.708-0.933	<0.001	0.829	0.750	0.829	0.750

### 3.7 Diagnostic value of miR-9-5p levels in peripheral blood mononuclear cells for outcomes in patients with sepsis

**Figure 10:**
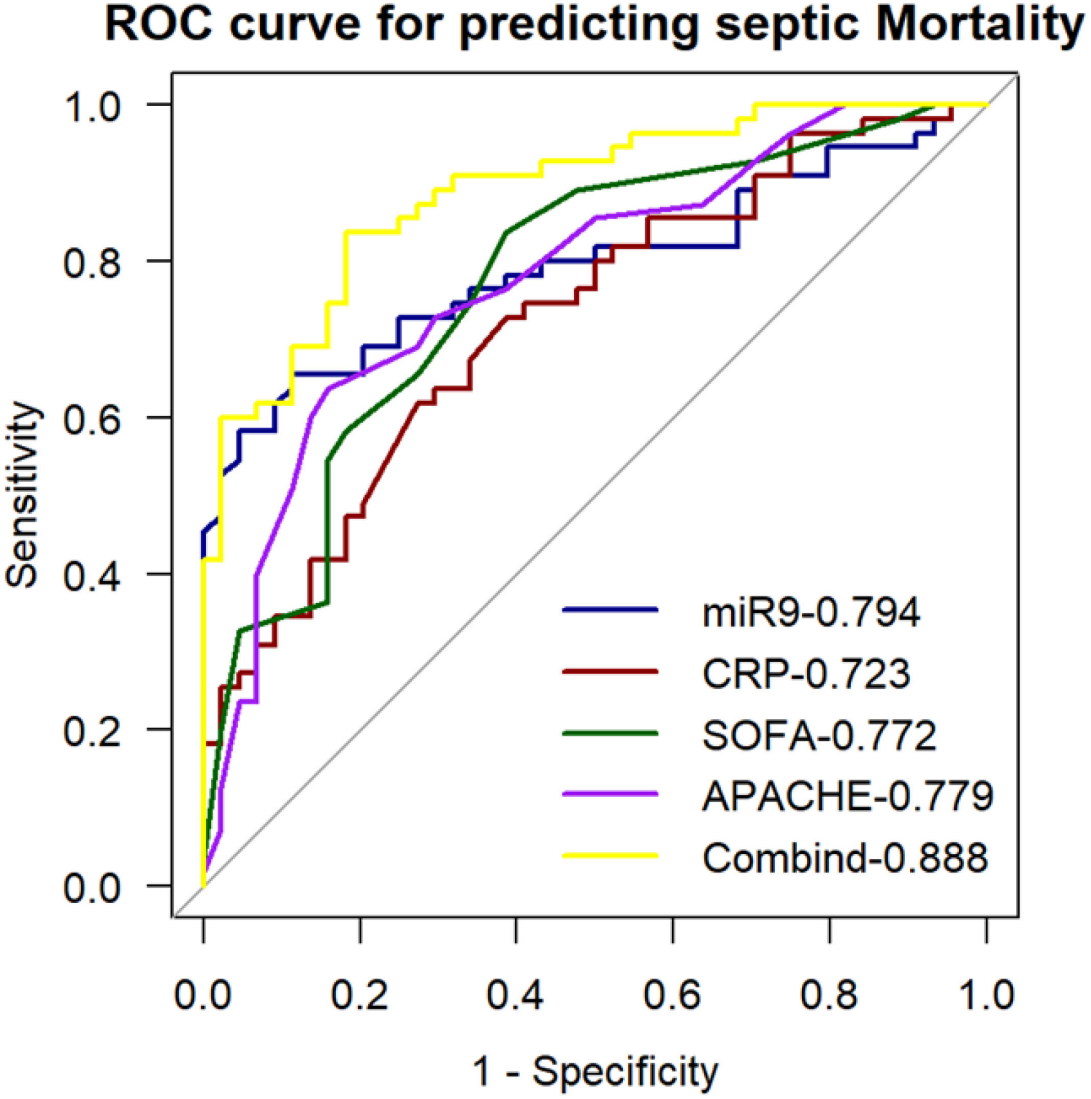
ROC curve for predicting sepsis mortality with the expression level of microRNA-9-5p in peripheral blood mononuclear cells.

We generated ROC curves for the miR-9-5p levels to explore their prediction accuracy for in-hospital death among patients with sepsis in ICU (**Figure 10**). The AUC was 0.794 at 95% confidence interval, 0.705-0.883. Further analysis was conducted to determine whether clinically feasible measures contributed to the prediction of mortality in patients with sepsis. The calculated AUC values for the SOFA score, APACHE II score, and CRP were 0.772 (95% CI: 0.679-0.866), 0.779 (95% CI: 0.688-0.872), and 0.723 (95% CI: 0.625-0.823). Predictive value of MiR-9-5p for mortality in patients with sepsis is equivalent to that of the SOFA score and APACHE II score. To develop a more accurate evaluation system, we constructed a combined predictive model incorporating all indicators using logistic regression analysis. The combined model yielded an AUC of 0.888, and its predictive accuracy was significantly improved compared to that of miR-9-5p alone (p < 0.05).

## 4 Discussion

Sepsis is among the most serious diseases worldwide and a major cause of mortality in critically ill patients in ICUs (Cecconi et al., 2018). According to the Sepsis 3.0 guidelines, sepsis is defined as the dysregulated response to infection that leads to life-threatening organ dysfunction as its major determinant. Current studies suggest that immune disorders underlie the pathogenesis of sepsis development (van der Poll et al., 2017; van der Poll et al., 2021). Current sepsis research primarily focuses on developing advanced diagnostic tools, refining classification systems, and implementing personalized treatment strategies based on molecular and biochemical profiles (Singer et al., 2016; Komorowski et al., 2022; Sinha et al., 2023). Macrophages have regulate the immune response during sepsis. Macrophages are among the first cell types that are activated upon pathogen invasion. They are highly heterogeneous and plastic, allowing a transition between the classically activated pro-inflammatory phenotype (M1, mostly induced by TLR ligands such as LPS and IFN-γ) and the alternatively activated anti-inflammatory phenotype (M2, mostly induced by IL-4 and IL-13) (Liu et al., 2014). Abnormal activation of macrophages predicts poor prognosis of diseases (Karakike and Giamarellos-Bourboulis, 2019). Available data indicates that microRNAs (miRNAs) are involved in the development and progression of sepsis (Formosa et al., 2022). MiRNAs are small endogenous RNA molecules that regulate gene expression at the post-transcriptional level (Lu and Rothenberg, 2018). Their small size, simple structure, high stability, and specificity render them easily detectable rapidly and accurately (Rupaimoole and Slack, 2017). The biogenesis of miRNA begins in the nucleus followed by transport out of cells by exosomes and other mechanisms and later transformed into mature molecules that degrade or silence mRNA (Bushati and Cohen, 2007). Compared with conventional biomarkers such as C-reactive protein (CRP) and procalcitonin (PCT), miRNAs offer several advantages: they not only increase early and remain persistently elevated during sepsis, but also exhibit remarkable stability in stored samples (Bindayna, 2024). Recent research has found that several miRNAs, including but not limited to miR-15a, miR-34a, and miR-27a, poses high diagnosis and prognosis of sepsis in adults and children (Wang et al., 2012; Abdelaleem et al., 2022; Ou et al., 2022). MiRNAs can also differentiate between sepsis patients with and without organ dysfunction. For example, miR-452-3p and miR-22-3p can diagnose sepsis kidney injury (Liu et al., 2020; Zhang et al., 2021), while miR-21 and miR-127-5p exert protective effects in sepsis-related lung injury (Zheng et al., 2023; Zhao et al., 2024). Accumulating evidence has shown that miRNA, such as miR-155, miR-146, miR-223, and let-7, participates in the pathophysiology of sepsis. These miRNAs interact with various components of the TLR-MYD88 signaling pathway, playing a role in immune regulation during sepsis (Ma et al., 2011; Nahid et al., 2011). Other studies have shown that miR-146a, miR-125b, and miR-124 directly target and regulate the expression of IL-1 and IL-6 (Saba et al., 2014; Ohnuma et al., 2019).

Studies have explored the role of circulating miRNAs to serve as sepsis markers (Bindayna, 2024). However, there has been less focus on the expression of these miRNAs in macrophages. In this study, we identified that miR-9-5p was upregulated in LPS-treated macrophages derived from both humans and mice, as confirmed by data from the GEO database and available literature (Lu et al., 2016). In a prospective COVID-19 cohort study, significant dysregulation of miR-199a-5p/miR-9-5p was observed in bronchial aspirate (BAS) samples, with an AUC as high as 0.80 for predicting in-ICU mortality (Molinero et al., 2021). The Smad2/miR-9/ANO1 loop has been confirmed to regulate inflammation in mouse sepsis model induced by LPS, knockdown of miR-9 attenuated the induced effects of LPS on IL-6 and TNF-α secretion (Zhen et al., 2019). Bioinformatic analysis demonstrated that miR-9-5p was the most differentially expressed miRNA between M1 and M2 polarized macrophages (Lu et al., 2016). SIRT1 is a member of the sirtuin family and plays a critical role in inflammatory diseases. As a target gene of miR-9-5p, its marked downregulation in sepsis promotes macrophage polarization toward the M1 phenotype, thereby exacerbating inflammatory injury (Ma et al., 2021). As research into cell death deepens, investigators have found that miR-9-5p alleviates sepsis-induced neuronal ferroptosis by inhibiting the expression of TFRC and GOT1, thereby mitigating sepsis-associated encephalopathy (Wei et al., 2022). Therefore, we explored whether the expression level of miR-9-5p in macrophages could reflect immune status in sepsis, serve as an additional diagnostic marker for sepsis severity, and potentially predict patient prognosis. This has yet to be validated in studies involving patient samples. We thus analyzed the qRT-PCR results of miR-9-5p mRNA levels in PBMCs from the patients with infection but non-sepsis, sepsis patients, septic shock patients, and healthy individuals. There were significant up-regulations of miR-9-5p mRNA levels in the PBMCs of sepsis and septic shock patients than patients infected with non-sepsis infections. Reports indicate that different bacterial infections induce distinct miRNAs in macrophages. For instance, let-7d, miR-15b, and miR-16 are differentially expressed in response to Gram-negative bacterial infections (Wu et al., 2013), whereas miR-133a and miR-668 are associated with Gram-positive bacterial infections (Hsieh et al., 2013). In contrast, we found that the expression levels of miR-9-5p did not differ between infection sites and pathogen culture results. These preliminary findings suggest that miR-9-5p may primarily reflect the intensity of the immune response or the severity of the condition induced by infection, rather than the presence of a specific pathogen. However, given the limited sample size of the present study, we plan to increase the sample size in future research and collect more detailed information on the infectious pathogens and sites of infection, enabling comprehensive stratified and subgroup analyses.

ROC and AUC analyses demonstrated that miR-9-5p expression in PBMCs effectively discriminated between infection and sepsis. Correlation analysis revealed that miR-9-5p was significantly associated with infection and organ injury, with a particularly high correlation coefficient of 0.655 with the Sequential Organ Failure Assessment (SOFA) score. We observed that as the number of dysfunctional organs and SOFA scores increased, the expression levels of miR-9-5p also rose significantly. PCT and CRP are classical biomarkers for the diagnosis and prognosis of sepsis, playing a crucial role in guiding clinical treatment (Zhou et al., 2024). Studies have suggested that PCT reflects sepsis severity more accurately than CRP (Tyagi et al., 2024), which is consistent with our findings in predicting 28-day mortality in sepsis patients. Researchers have demonstrated that the combination of CRP and PCT enhances predictive performance. However, in our study, the predictive efficacy of miR-9-5p combined with CRP and PCT was not significantly superior to that of miR-9-5p alone, suggesting its feasibility as a diagnostic biomarker for sepsis. The miR-9-5p expression level in PBMC may serve as a diagnosis marker of septic shock and can predict in-hospital mortality among sepsis patients. To further verify the validity of our results, we established a CLP-induced mouse model. This study has some limitations that should be acknowledged. Firstly, the patient sample size was limited and derived from a single center, which may affect the generalizability of the findings. Additionally, although we excluded underlying diseases that could potentially affect the patients’ immune status, we did not fully account for the potential impact of glucocorticoids and other treatments on immune function. To address these issues, future studies will aim to expand the sample size through multicenter and prospective studies and establish stricter inclusion criteria to enhance the representativeness and applicability of the results. Secondly, this study did not monitor miR-9-5p expression levels dynamically, preventing an understanding of its temporal changes during sepsis progression. Future research will include longitudinal follow-up studies to investigate the time-dependent expression patterns of miR-9-5p in sepsis. Additionally, the potential regulatory functions and molecular mechanisms of miR-9-5p in sepsis were not thoroughly explored in this study. To further elucidate its role, future studies will focus on deciphering the downstream signaling pathways involved in miR-9-5p-mediated immune regulation.

## 5 Conclusion

The relative expression level of miR-9-5p in PBMCs may serve as a diagnostic marker with good sensitivity and specificity for sepsis and septic shock. It can also reflect the degree of severity and prognosis of sepsis.

## Data Availability

The original contributions presented in the study are included in the article/**Supplementary Material**. Further inquiries can be directed to the corresponding author/s.
